# Beyond the blood-brain barrier: humanised mice, the missing link in glioblastoma research

**DOI:** 10.1038/s41388-026-03989-1

**Published:** 2026-09-20

**Authors:** Reza Shirazi Nia, Jian Lu, Daniel De Vega, Niloufar Poudine, Chaoyue Pan, Fengqi Zhou, Jianxiong Ji, Georgios Giamas

**Affiliations:** 1 International Oncology Institute, The First Affiliated Hospital of Zhejiang Chinese Medical University, Oncology Department of the First Affiliated Hospital of Zhejiang Chinese Medical University, Hangzhou, China; 2 Department of Neurosurgery, The Second Affiliated Hospital of Zhejiang University School of Medicine, Zhejiang University, Hangzhou, China; 3 Zhejiang Key Laboratory of Research and Transformation for Major Neurosurgical Diseases, Hangzhou, China; 4 Department of Biochemistry and Biomedicine, School of Life Sciences, University of Sussex, Brighton, UK

**Keywords:** CNS cancer, Cancer models

## Abstract

Glioblastoma (GBM) remains a major challenge in neuro-oncology, associated with a high rate of mortality despite decades of intensive research and therapeutic advancements, underscoring the urgent need for innovative preclinical platforms that can more accurately recapitulate the biological and pathological features of human disease. While conventional animal models have contributed to our understanding of GBM biology and the evaluation of treatment efficacy, they fail to capture the full complexity and heterogeneity of the tumour microenvironment (TME). Ex vivo models are associated with certain advantages in this context; however, they can not mirror the complex dynamic and multicellular interactions present in living organisms, particularly the critical treatment barriers unique to the central nervous system: the blood-brain barrier (BBB), blood-cerebrospinal fluid barrier (BCSFB) and blood-meningeal barrier (BMB). In response to these limitations, humanised mouse models have emerged as an advanced platform capable of faithfully mimicking the molecular, pathological and immunological features of human GBM. These models enable the replication of complex in vivo crosstalk between the immune system and the TME, while preserving the relevant treatment barriers that govern drug delivery to the brain. Accumulating evidence indicates that humanised mouse models closely reproduce the infiltration of human immune components into the TME, enabling the study of clinically relevant interactions that contribute to therapeutic resistance and treatment failure in GBM. This review aims to provide a comprehensive and systematic overview of the currently employed humanised mouse models in GBM research, highlighting their applications and comparative advantages. Finally, we evaluate the opportunities and challenges associated with each model and discuss future directions to increase the translational relevance and predictive power of preclinical GBM research.

**Figure:**
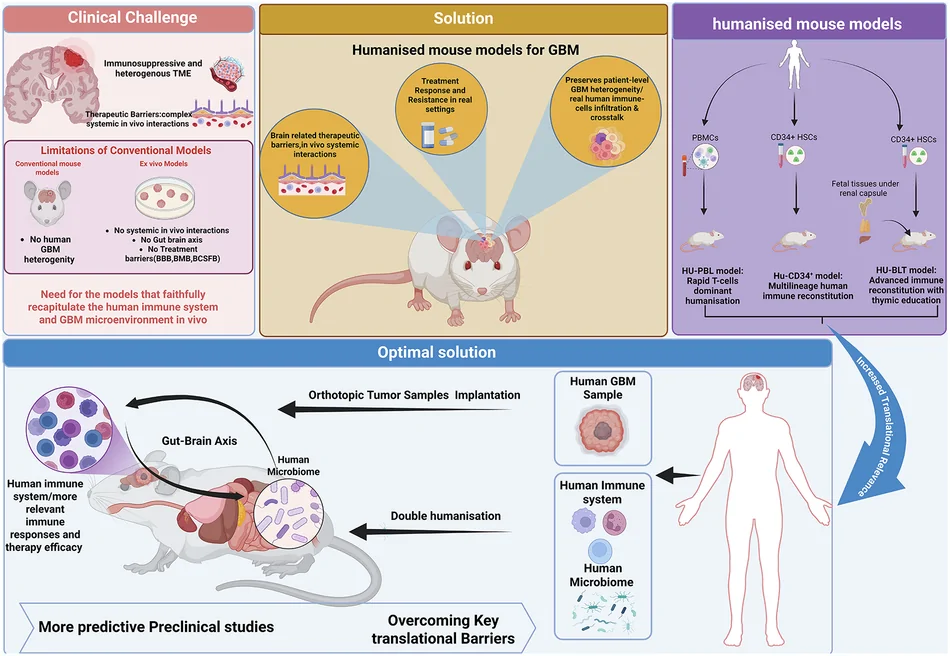
Humanised mouse models provide a valuable translational platform combining the human immune system and PDX orthotopic engraftment. Compared to conventional models and ex vivo models, these models can reproduce the complex cross-talk between tumour cells and the immune system, tumour heterogeneity, immunosuppressive TME, as well as complex in vivo interactions such as brain-specific barriers, including BBB, BCSFB and BMB. Future implementation of the human gut microbiome in these models has the potential to further increase translational relevance and precision in GBM research. Created in BioRender.com.

Humanised mouse models provide a valuable translational platform combining the human immune system and PDX orthotopic engraftment. Compared to conventional models and ex vivo models, these models can reproduce the complex cross-talk between tumour cells and the immune system, tumour heterogeneity, immunosuppressive TME, as well as complex in vivo interactions such as brain-specific barriers, including BBB, BCSFB and BMB. Future implementation of the human gut microbiome in these models has the potential to further increase translational relevance and precision in GBM research. Created in BioRender.com.

## Introduction

Glioblastoma (GBM) is the most common and lethal primary brain tumour in adults, with an annual incidence of three cases per 100,000 [1]. Despite rapid research advances, prognosis remains extremely poor and the disease is considered incurable. In response, immunotherapies have emerged as a paradigm shift in GBM and many other cancers [2], holding clinical promise by overcoming immune resistance and enhancing treatment efficacy [3]. Nevertheless, although some clinical trials have reported clinical efficacy in GBM, patient responses remain highly variable and substantial knowledge gaps persist. This variability underscores the critical need for reliable research methodologies to further elucidate the biological mechanisms underlying GBM progression and treatment response [4]. A major reason for this variability is the GBM tumour microenvironment (TME) itself. Human GBM is considered an immunologically cold, myeloid-dominated tumour in which tumour-associated macrophages constitute about 30% of the tumour mass and 80% of the immune cells. Additionally, tumour-associated lymphocytes populate the TME to a much lesser extent than monocytes. T cells represent about 0.9% of all cells and 10–15% of the immune fraction in GBM, where they frequently dysfunctional due to the ignorance, exhaustion, anergy, tolerance and senescence, failing to mount an effective immune reaction [5, 6] also GBM microenvironment can induce the systemic immunosuppression partly through downregulation of sphingosine-1-phosphate receptor-1 which is a crucial mediator for the T-cell egress from the thymus and secondary lymphoid organs leading to the sequestering naïve T cells within the bone marrow and leading to reduced efficacy of the T-cells activating therapies [7, 8]. The standard of care for GBM includes surgical resection as well as radiotherapy and chemotherapy with Temozolomide [9]. Although this protocol induces local and systemic immune effects, it fails to induce long-lasting immune activation; additionally, radiotherapy induces acute lymphopenia that is sustained through the adjuvant temozolomide therapy, leading to further systemic immunosuppression. Also, corticosteroids are used commonly in GBM patients to alleviate the symptoms of cerebral vasogenic oedema; their usage is associated with more severe acute lymphopenia and lower overall survival [10]. These observations explain why many treatments show limited efficacy in GBM patients.

**Fig. 1:**
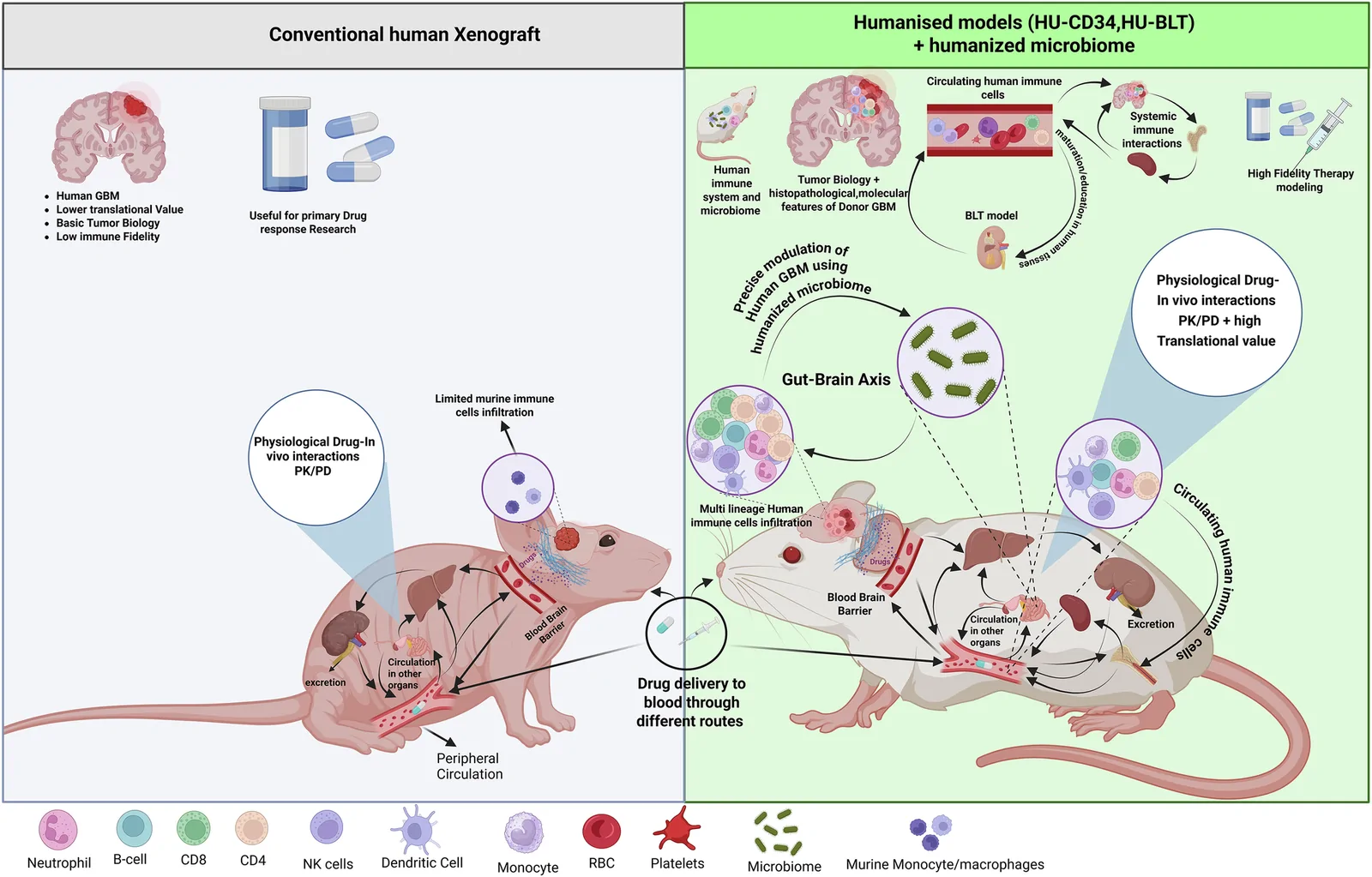
Humanised GBM mouse models capture immune interactions absent in the conventional xenografts. The conventional animal models provide the in vivo platform for drug testing in GBM, including pharmacokinetic/Pharmacodynamic interactions, metabolism and excretion as well as treatment barriers such as the BBB, BCSFB and BMB with a good relevance for GBM treatment, but they lack the heterogeneity we see in the human GBM, this underscores importance of the humanised mouse models with the infiltrating and circulating immune cells, systemic immune interactions and at the same time the physio-pathologic drug pharmacokinetic interactions offering high validity and precision. Implementing the human gut microbiome in the future of these models can increase the translational relevance and precision in these models. Abbreviations: BBB Blood-brain barrier, BMB blood-meningeal barrier, BCSFB blood-cerebrospinal fluid barrier, PK pharmacokinetic, PD Pharmacodynamic. Created in BioRender.com.

Mouse models are indispensable tools in GBM research [11]; however, when considering the inherent complexity of the TME, conventional models often fail to fully recapitulate its multifaceted nature [12]. Ex vivo systems, such as organoids and patient-derived tumour fragments, are useful for studying TME components, yet they lack the systemic physiological interactions and multicellular dynamics present in living organisms, including critical features such as blood-brain barrier (BBB) penetration and its associated complications in human GBM [13, 14]. The BBB filters ~98% of small molecules and virtually all large molecules (e.g. antibodies and growth factors). It is not uniform; it shows regional heterogeneity, remaining intact in aggressive tumour regions while disrupted in others [15]. Another key barrier is the blood–cerebrospinal fluid barrier (BCSFB), located at the choroid plexus epithelium where cerebrospinal fluid (CSF)-producing epithelial cells are connected by tight junctions and supplied by fenestrated capillaries [16]. In addition, the blood–meningeal barrier (BMB) plays an important role in GBM treatment [17]. Therapeutic agents must overcome these barriers to effectively reach the brain and tumour. In addition to these factors, emerging evidence suggests that the gut microbiome influences GBM growth, progression and even temozolomide efficacy via the gut-brain axis, highlighting the complex multimodal nature of GBM and likely other cancers as well [18, 19]. Taken together, these treatment-related challenges have spurred growing interest in the development and application of humanised mouse models that recapitulate the human immune system and tumour-immune interactions within the complexity of an in vivo system. (Fig. 1). Humanised mouse models are immunodeficient mice engrafted with key components of the human immune system, specifically designed to address these obstacles. When such humanised mice are engrafted orthotopically with the human tumour samples, they can faithfully model the intricate interactions between the tumour and the human immune system. Humanised GBM mouse models facilitate the translational pipeline from bench to clinical trials, improving the precision of therapeutic development; they recapitulate at a highly acceptable rate the histopathologic, molecular, vascular and TME features of their corresponding organoid or tumour tissues. Consequently, they rank among the most powerful tools currently available for the study of GBM [20]. It is noteworthy that humanised models discussed in this review reconstitute the human haematopoietic compartments; they do not humanise the host central nervous system. BBB, BCSFB and BMB remain of murine nature. Therefore, the translational advantage of orthotopic humanised models lies in situating a human tumour and a human immune system within an anatomically appropriate in vivo CNS compartment.

## Overview of humanised mouse models

The evolution of humanised mouse models started with the implantation of human tumours in athymic nude mice (Foxn1^nu^). A major milestone in this field was the knock-out(KO) of the common γ-chain of the Interleukin(IL)-2 receptor (IL-2Rγ), a subunit shared by receptors for IL-2, IL-4, IL-7, IL-9, IL-15 and IL-21. This genetic modification resulted in the loss of functional murine natural killer (NK) cells. Subsequently, combining the IL-2Rγ_c_ KO with either the scid mutation or Rag1 and Rag2 KOs led to the development of next-generation immunodeficient strains, including NSG (NOD.Cg-Prkdc^scid^ Il2rg^tm1Wjl^/SzJ), NOG (NOD.Cg-Prkdc^scid^ Il2rg^tm1Sug^/JicTac) and BRG (BALB/c Rag2^–/–^ IL-2Rg_c_^–/–^) mice [21]. To enhance human immune engraftment and myeloid differentiation, key human cytokines have been introduced transiently or via targeted knock-ins across various strains, leading to the establishment of strains such as NSG-SGM3, NOG-EXL, BRGST, MISTRG, MISTRG6, MITRG,hIL2-NOG, SRG-15, IL-15 NOG and NSG-Tg(hu-IL15), all of which incorporate knock-ins of the human immune components [21–23].

**Fig. 2:**
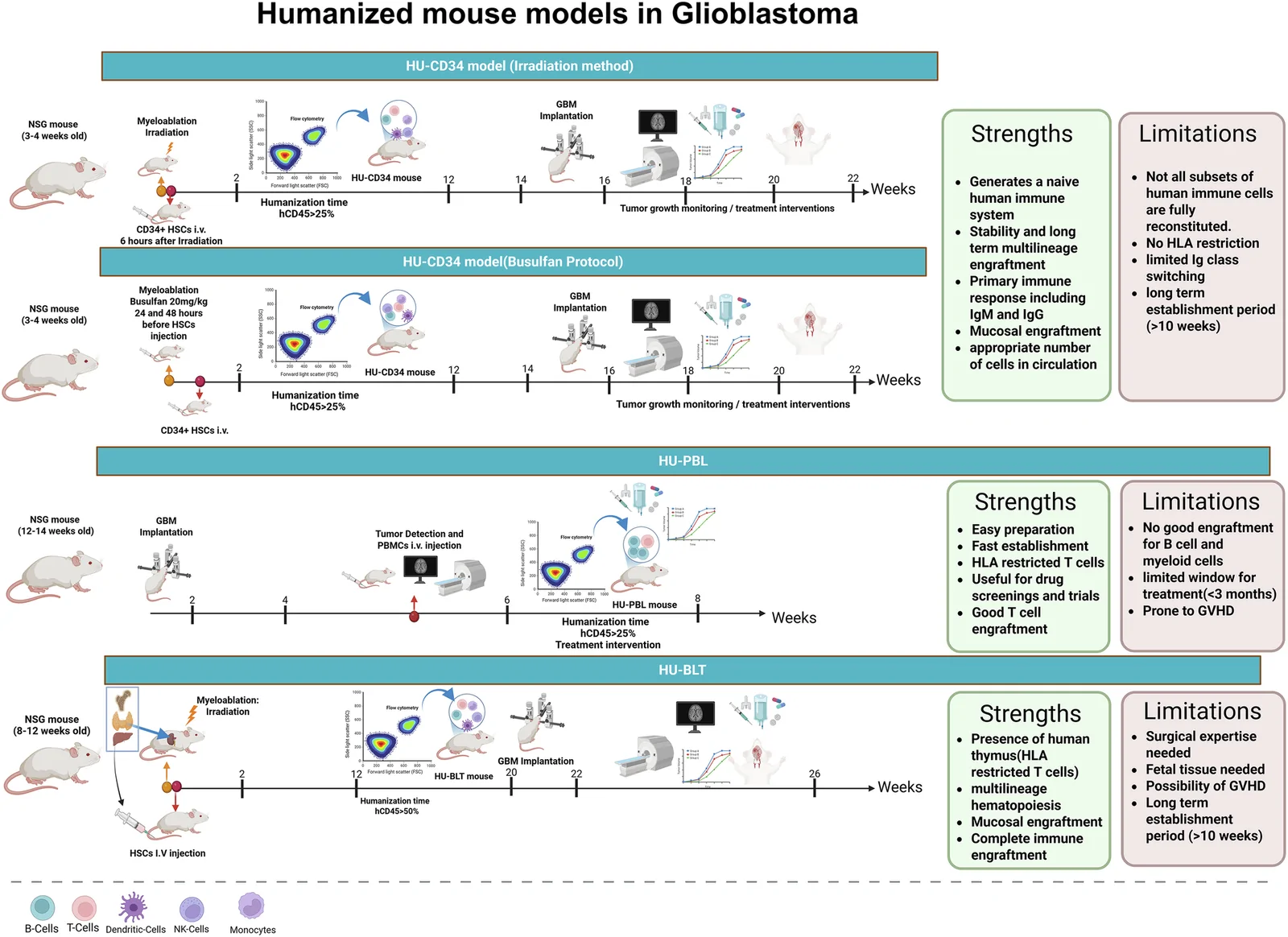
Main types of humanisations used in the GBM and their ability to establish different lineages of human immunity as well as the strengths and limitations of each model. The Hu-PBL model is an easy and cheaper way of humanisation, it is a strong way to study T-cell functions, the Hu-CD34^+^ model gives a multi-lineage establishment but it needs a long time for engraftment and it lacks the human leukocyte antigen (HLA) restriction, the Hu-BLT models is the most complete model giving the closest simulation to human immune system but it has complications of sourcing the fetal tissues; abbreviations: GVHD, graft versus host disease; HLA, human leukocyte antigen;hCD45^+^, Human CD45 positive cells. Created in BioRender.com [2, 21, 23, 31, 41, 43, 44].

In the context of GBM research, two principal sources of human immune cells are used to generate three distinct types of humanised mouse models: human peripheral blood mononuclear cells (PBMCs) (giving rise to the Hu-PBL model) and hematopoietic stem cells (HSCs) (leading to the Hu-CD34^+^ model and the human bone marrow, liver and thymus (Hu-BLT) model) (Fig. 2). Following the establishment of the human immune system, these humanised mice are co-engrafted with human GBM samples or cells. Additionally, some GBM studies use humanised microbiome (HUM) mice in which the murine gut is colonised with human microbiota before intracranial engraftment; here, the immune system remains of mouse origin [19].

### Hu-PBL model: rapid T-cell-dominant humanisation

In the Hu-PBL model, PBMCs are intravenously, intraperitoneally, or intrasplenically inoculated into immunodeficient mice. PBMCs are typically isolated from circulating blood and occasionally from lymph nodes or the spleen of healthy donors who are partially major histocompatibility complex (MHC) matched to the subsequent tumour graft, thereby introducing intrinsic allogenicity [21, 24]. Notably, no prior irradiation or myeloablation is needed for this model [25]. Within 4 weeks of inoculating 2 × 10^7^ PBMCs into NSG mice, human CD45^+^ cells reach ~50% of circulating leukocytes, predominantly CD3^+^ T lymphocytes, making the Hu-PBL model suitable for studying T-cell-directed therapies [23]. The principal limitation of this model is lethal graft-versus-host disease (GVHD), the severity and onset of which correlate with CD4^+^ T-cell engraftment [26]. Prior irradiation may accelerate GVHD onset, thereby restricting the therapeutic observation window to ~4-6 weeks following PBMCs injection [27, 28]. To mitigate the GVHD risk, which is mediated by murine MHC molecules, strains with inactivated MHC class I and II genes have been developed, including the NSG-MHC-DKO and NOG-dKO models. These strains exhibit limited GVHD occurrence and are therefore particularly useful for Hu-PBL-based studies [29–31]. Despite these challenges, the Hu-PBL model remains an economical and rapid approach, representing its primary advantages.

### Hu-CD34+ model: multi-lineage human immune reconstitution

The Hu-CD34⁺ model is established by intravenous, intrafemoral, or intraperitoneal injection of human CD34^+^ HSCs into neonatal or adult immunodeficient mice. Human HSCs may be isolated from umbilical cord, granulocyte colony-stimulating factor (G-CSF)-mobilised PBMCs, fetal liver, or adult bone marrow [21, 23]. It has been reported that HSC inoculation into newborn or young mice (up to 4 weeks of age) accelerates T-cell development [32]. This model requires preconditioning -typically sublethal irradiation- to ablate murine HSCs and enhance human cell engraftment; alternative approaches include administration of busulfan or antibody-mediated deletion of mouse progenitor cells can be used [33, 34]. The Hu-CD34⁺ model supports multi-lineage human hematopoiesis, enabling engraftment of both innate and adaptive human immune cell populations [35]. However, several limitations have been noted: the majority of human B-cells are immature CD5^+^ B-cells; both T-cells and NK cells exhibit partial functional impairments; and monocytes display varying degrees of phenotypic immaturity [21]. Additionally, this model requires a longer timeframe for establishment compared to the Hu-PBL model. A key advantage is that GVHD occurs only rarely in Hu-CD34⁺ mice [23].

### Hu-BLT model: advanced immune reconstitution with thymic education

The Hu-BLT model is established by intravenous or intraperitoneal injection of human CD34^+^ HSCs into immunodeficient mice, combined with surgical implantation of autologous human fetal liver and thymic tissues beneath the mouse kidney capsule. This approach supports the development of a multi-lineage hematopoietic population such as T-cells, B-cells, dendritic cells (DCs), macrophages and myeloid cells, in addition to diverse T-cell subsets such as CD4^+^, CD8^+^ and regulatory T (Treg) cells [23, 36, 37]. Unlike the Hu-CD34^+^ model, the Hu-BLT model enables T-cell maturation within the transplanted human thymus tissue, thereby facilitating authentic thymic education [38]. To date, the Hu-BLT model represents the most complex humanised mouse system. Nevertheless, it presents several challenges, including the requirement for survival surgery and limited accessibility of human fetal tissues. Furthermore, compared with the Hu-CD34^+^ model, the Hu-BLT model exhibits a higher incidence of GVHD at a greater rate, which results in a shortened lifespan. Of note, a modified Hu-BLT mouse model generated on a C57BL/6 Rag2_null_ IL2rg^null^Cd47^null^ background does not develop GVHD and is suitable for long-term studies [2].

## Humanised mouse models in GBM: current applications

One of the central challenges in neuro-oncology is the complexity and heterogeneity of GBM. Although patient-derived xenograft (PDX) murine models are widely developed and utilised in GBM research, they fail to fully capture the complex immunologic interactions within the TME [39]. Humanised mouse models have emerged as valuable platforms for testing and validating a broad range of immunotherapies, including antibodies, adoptive cell therapies, oncolytic viruses, small molecule inhibitors and agonists. Furthermore, first-generation immune checkpoint inhibitors targeting the PD-1/PD-L1 axis, CTLA4, or LAG3 have been studied using humanised mouse models. Notably, humanised mouse models are beneficial tools to study chimeric antigen receptor (CAR) T-cell therapies in GBM; for instance, humanised NOG-EXL mice reconstituted with human CD34⁺ HSCs and orthotopically engrafted with patient-derived glioma stem cells were recently used to demonstrate that GPNMB-directed CAR-T cells deplete both tumour cells and immunosuppressive myeloid populations [40]. In addition, humanised mouse models are increasingly employed in the study of combination immunotherapies [2].

### Comparative evaluation of Hu-CD34^+^ and Hu-PBL models

One of the most recent comprehensive studies using two humanised mouse models of GBM was performed by Moreno-Sanchez et al. (Unpublished preprint) [41]. The authors established Hu-CD34^+^ and Hu-PBL models in the NSG(NOD.Cg-Prkdc^scid^ Il2rg^tm1Wjll^/SzJ) mouse strain. Key findings included a preserved baseline brain immune landscape in immunocompromised strains and conserved microglia–GBM crosstalk in PDOX models. Tumours grew and were grafted in both humanised models with histopathology similar to non-humanised NSG mice, suggesting that human immune reconstitution, regardless of HLA compatibility, does not inhibit tumour growth. Moreover, in the Hu-CD34^+^ model, there was a higher number of hCD45^+^ cells compared to Hu-PBL in the bone marrow, spleen and blood samples. Both tumour-bearing models showed splenomegaly and increased hCD45^+^ counts compared with the humanised healthy controls, reflecting tumour-induced immune activation and systemic modulation by GBM. Both models resulted in full human immune reconstitution in the circulation; however, the Hu-CD34^+^ model exhibited higher B-cell reconstitution in blood and spleen, whereas the Hu-PBL model was mainly restricted to T-cells. For local TME reconstitution, both models showed higher hCD45^+^ infiltration in the tumour than in the contralateral hemisphere. Hu-PBL yielded more CD4⁺ T-cells, whereas Hu-CD34⁺ had more monocytes and DCs, indicating distinct reconstitution patterns. Overall, these two models supported the establishment of organised immune niches resembling those observed in human GBM. Moreover, spatial distribution analysis of CD4 + T-cells and myeloid subsets revealed that both models effectively recapitulate the immunosuppressive nature of GBM, as evidenced by predominant myeloid cell activity and limited T-cell infiltration. In agreement with the above findings, Jun Takei et al. investigated GBM tumour profiles and immune cell infiltration using flow cytometry, immunohistochemistry and single-cell RNA sequencing in two humanised GBM mouse strains -NSG-SGM3 and NOG-EXL strains- humanised via CD34^+^ cell inoculation and xenografted with irradiation-resistant GBM samples. Their results demonstrated a wide range of infiltrating human immune cells, including T-cells, NK cells and myeloid lineage cells, within PDX tumours. Moreover, the gene expression profiles of these infiltrating cells closely mirrored those observed in recurrent human GBM. Similar to human GBM, these models exhibited increased tumour heterogeneity, characterised by an elevated proportion of neural progenitor-like tumour cells [42].

### Advanced humanisation models: Hu-BLT and HLA-restricted strains

In one study, 8–12-week-old NSG mice were used to establish the Hu-BLT model. Mice were first subjected to sublethal irradiation (2 Gy), followed by kidney-capsule implantation of human fetal thymus and liver (17–21 weeks gestation). Within 6 hours of tissue engraftment, 1 × 10⁵ autologous fetal liver-derived human CD34⁺ HSCs were administered intravenously. Animals were monitored for 12–20 weeks; human immune system reconstitution was defined as >50% human CD45⁺ cells within the lymphocyte gate, with >40% of these cells expressing human CD3⁺. Subsequently, Hu-BLT mice were engrafted with mouse-passaged dissociated GBM cells. The results demonstrated infiltration of human immune cells into intracranially established GBMs and into the immune compartments of other tissues. Multiplex immunofluorescence revealed that the TME was dominated by myeloid cells, with a smaller T lymphoid component mirroring the immunosuppressive nature of GBM [43]. Although NSG remains the most common strain, others are useful. DRAG mice are NRG mice transgenic for human HLA-DR4, lacking mature T, B and NK cells. In a study by Rashmi Srivastava et al., the human immune system was established in DRAG mice by irradiation at a dose of 250 cGy followed by intravenous injection of 2 × 10⁵ CD34^+^ hDR4⁺ HSCs six hours post-irradiation. Mice were then engrafted with 2 × 10^5^ GBM patient–derived tumor sphere cells. In this model, engrafted tumours faithfully recapitulated the pathological characteristics and immune cell populations observed in human GBM. Treatment of tumour-bearing humanised mice with anti-human PD-1 antibodies reduced major tumour-infiltrating immunosuppressive populations such as CD4^+^PD-1^+^ and CD8^+^PD-1^+^ T-cells, CD11b^+^CD14^+^HLA-DR^+^ macrophages, CD11b^+^CD14^+^HLA-DR^−^CD15^−^ and CD11b^+^CD14^−^CD15^+^ myeloid-derived suppressor cells. These findings position the humanised DRAG model as an important model for evaluating immunotherapy efficacy in GBM research [44].

### Emerging applications of humanised GBM models

In a busulfan-based Hu-CD34^+^ humanised model using the NOG strain, Brain Tumour stem cells (BTSCs) were engrafted to evaluate CD133 mRNA-loaded DCs vaccination as a novel therapy for GBM. The results demonstrated concurrent activation of CD8⁺ and CD4⁺ T-cells, leading to a potent and durable immune response. This response was associated with suppression of CD133⁺ glioma stem cell propagation and consequent inhibition of tumour growth [45]. In a preliminary study reported in conference abstract form, Binder et al. showed that intraosseous injection of patient-derived CD34⁺ HSCs (from bone marrow at the time of resection), followed by intracranial implantation of patient-matched GBM organoids into NOG-EXL mice, resulted in CD45⁺/CD33⁺ infiltration in proximity to the tumour. Potentially enabling personalised medicine strategies tailored to individual tumour characteristics [46]. Using positron emission tomography (PET), researchers employed a Hu-PBL model engrafted with patient-derived GBM to directly visualise tumour-infiltrating human T-cells [47]. The same group non-invasively quantified human CD4⁺ cells with immunoPET radiotracers [48], showing that these models can monitor treatment effects on the human GBM TME. In another study, Hu-BLT was established in an NSG-SGM3 strain. After tumour (PDX, U87) engraftment and human T-cell-depleting antibody treatment, human T-cells were confirmed in engrafted GBM but absent in the tumour-free hemisphere and CD4^+^ and CD8^+^ T-cells were detected among CD3^+^ GBM-infiltrating T-cells, with increased numbers of CD3^+^ and CD4^+^ cells associated with markers of immunosuppressive Tregs [49].

In a study using the NOG-dKO Hu-PBL model, the efficacy of the STAT3 inhibitor STX-0119 was evaluated in a temozolomide-resistant U87 model. STAT3 inhibition resulted in pronounced tumour growth inhibition, accompanied by accumulation of tumour-infiltrating lymphocytes and macrophages in tumour samples [50]. A summary of the humanised mouse models used in GBM research is provided in Table 1A**;** additionally, a category level of the advantages and limitations of each model is presented in Table 1B.

**Table 1:** **A** Studies using humanised mouse models for GBM: **B** Comparative advantages and limitations of the main categories of humanised mouse models used in GBM research.

A						
Study	Mouse strain	Preconditioning/humanisation model	Injection Method and the number of cells	Cells engrafted successfully	GBM model used	Drugs
Moreno-Sanchez et al.^*^ [41]	NSG mice (NOD.Cg-Prkdc^scid^ Il2rg^tm1Wjll^/SzJ)	gamma irradiation 140cGY/Hu-CD34^+^	6 × 10^4^ CD34^+^HSCs/NA	B-cells, CD4^+^T-cells, CD8^+^ T-cells, NK cells, monocytes, DCs and neutrophils in bone marrow, blood and spleen samples (low levels of neutrophils in blood samples)	Intracranial GBM organoids	pembrolizumab (anti-PD-1), ragifilimab (anti-GITR) and combination treatment
		20 mg/kg of busulfan 48 h and 24 h before HSCs inoculation I.V./Hu-CD34^+^	5–10 × 10^4^ CD34^+^ HSCs/ intravenously			
		NO conditioning for Hu-PBL	10^7^PBMCs/intravenously	B-cells, CD4^+^T-cells, CD8^+^T-cells and variable /low numbers of NK cells, monocytes, DCs and neutrophils in bone marrow, blood and spleen samples		
Jun Takei et al.[42]	NSG-SGM3 (NOD.Cg-Prkdc^scid^Il2rg^tm1Wjl^Tg(CMVIL3, CSF2, KITLG)1Eav/MloySzJ)), NOG-EXL (NOD.Cg-Prkdc^scid^ Il2rg^tm1Sug^Tg(SV40/HTLV-IL3,CSF2)10-7Jic/JicTac)	X-rays at 90 cGy/ Hu-CD34^+^	0.1 × 10^6^ CD34^+^HSCs/intravenously	B-cells(CD-19), CD4^+^T-cells, CD8^+^T-cells, NK cells(CD56), monocytes/ macrophages(CD14, granulocytes (CD66b) in blood and tumour samples (NOG-EXL showed significantly higher percentage of humanisation (%))	GBM PDX resistant to irradiation	NA
Longsha Liu et al. [43]	NSG(NOD/SCID/IL2Rγ − /−)	Sublethal irradiation at 2-Gy/Hu-BLT	1 × 10^5^ CD34+ HSCs/ intravenously	CD56 + NK cells, CD11c+ DCs, CD68+ macrophages and T lymphocytes infiltration in tumour samples and at variable levels in bone marrow, spleen and tumour draining lymph nodes	patient-derived GBM cells	NA
Rashmi Srivastava et al. [44]	DRAG (NOD.Rag1KO.IL2RγcKO)	gamma irradiation with a dose of 250 cGy/ Hu-CD34^+^	2 × 10^5^ hDR4+ HSCs/intravenously	human lymphocytes (CD4^+^ T-cells, CD8^+^ T-cells, NK cells and B-cells), CD14^+^ cells, CD11b and CD11c expressing cells in the PBMCs and spleens with variable numbers	GBM patient-derived tumorsphere	anti-PD-1 antibody
Angelique Sao-Mai Sy Do et al. [45]	NOG(NOD.Cg-Prkdc^scid^ Il2^rgtm1Sug^/JicTac mice)	Intraperitoneal injection of 25 mg/kg Busulfan/ Hu-CD34^+^	5 × 10^4^ CD34^+^ HSCs/intravenously	human CD3-, CD4-and CD8-positive cells in blood samples	Human Brain tumour stem cells	vaccination using modified CD133 mRNA
Zev Binder et al.^**^ [46]	NOG-EXL(hGM-CSF/hIL-3 NOG)	NA/Hu-CD34^+^	10^4^ of CD34+ HSCs/Intraosseous injection	human-derived CD45^+^/CD33^+^ cells observed in the proximity to the engraftment region	GBM Organoids derived from the same patient.	NA
Veronica L Nagle et al. [47]	NOG(NOD.Cg-Prkdc^scid^ Il2^rgtm1Sug^/JicTac)	NO Preconditioning/ Hu-PBL	10^7^ PBMCs/intravenously	Human CD8^+^ T-cells comprised and human CD3^+^ T-cells in mouse spleen samples	Patient-derived cell lines	NA
Veronica L Nagle et al. [48]	NOG (NOD.Cg-Prkdc^scid^ Il2^rgtm1Sug^/JicTac)	NO Pre conditioning/ Hu-PBL	10^7^ PBMCs/intravenously	Human CD4^+^ T-cell infiltration in GBM. including: Tregs (CD3^+^CD4^+^FOXP3^+^CD25^+^ cells/CD3^+^CD4^+^ cells) or activated conventional CD4^+^ T-cells including (CD3^+^CD4^+^FOXP3-CD45RA-CD45RO^+^ cells/CD3^+^CD4^+^ cells) with a minority of naïve CD4^+^ T-cells (CD3^+^CD4^+^FOXP3-CD45RA^+^CD45RO-/CD3^+^CD4^+^ cells)	GBM PDXs	NA
Lijie Zhai et al. [49]	NSG-SGM3(NOD.Cg-Prkdc^scid^ IL2^rgtm1^Wjl Tg (CMV-IL3, CSF2, KITLG)1Eav/MloySzJ)	NA/Hu-BLT model	NA	Presence of both CD4^+^ and CD8^+^ in the draining cervical lymph nodes (cLN) and spleen and tumour samples, markers expressing immunosuppressive Treg as well as CD3^−^CD45^+^ human myeloid cells in GBM samples	U87, PDX tumour cells	anti-human CD4, anti-human CD8 monoclonal antibodies
Yasuto Akiyama et al. [50]	NOG-dKO(MHC-double knockout mouse)	X-rays irradiation 2.5 Gy	1 × 10^7^ human PBMC /intravenously	CD3^+^,CD4^+^CD8^+^,CD25^+^FoxP3^+^,CD11b^+^CD33^+^,CD45RO^+^CD127^+^, CD3^+^PD-1^+^ cells were present in the tumour and spleen samples	TMZR U87 cells subcutaneously engrafted into the flank region	STX-0119(STAT3 inhibitor)
Elinor Willis et al. [76]	NSG-SGM3 (NOD. Cg-Prkdc^scid^ Il2rg^tm1Wjl^ Tg(CMV-IL3, CSF2, KITLG)1Eav/MloySzJ), NOG-EXL (NOD. Cg-Prkdc^scid^ Il2rg^tm1Sug^ Tg(SV40/HTLV-IL3,CSF2)10-7Jic/JicTac)	30 mg/kg intraperitoneal busulfan/Hu-CD34^+^	2 × 10^4^ human CD34^+^ HSCs/intraosseous	Both strains had a distribution of human B and T-cells in the spleen, with slightly higher distribution of T cells in NSG-SGM3. In the bone marrow of both strains, the human myeloid cells predominated, while B-cells and T-cells were present, with higher T-cells in NSG-SGM3. In peripheral blood, both strains had human lymphoid (CD3 + /CD19 + ) cells and human myeloid (CD33 + ) cells, which were higher in NSG-SGM3, especially for the human myeloid (CD33 + ) cells.	Organoids generated from the tumour	NA

B					
Model category	How it is generated	Human compartments reconstituted	Advantages	Limitations	Suitable GBM application
Hu-PBL	Intravenous/ intraperitoneal or intrasplenic injection of human PBMCs ~10^7^/mouse; no preconditioning required.	Predominantly mature T-cells (CD3⁺); variable and generally low B-cell, NK-cell, monocyte, Neutrophil and DCs reconstitution.	• Rapid engraftment. • Technically simple and least expensive. • Mature effector T-cells make it well suited to T-cell-directed therapies. • Autologous tumour–PBMC pairs are feasible in this model. • HLA-restricted T cells are available in this model.	• Lethal xenogeneic GVHD restricts the observation window to approximately 4–6 weeks. • Minimal human myeloid compartment is a drawback regarding the myeloid-dominated GBM TME.	Short-term drug screenings and trials related to T-cell-directed agents, immune checkpoint inhibitors, chimeric antigen receptor (CAR) therapies and bispecific monoclonal antibodies.
Hu-CD34⁺	Preconditioning (sublethal irradiation or busulfan administration) followed by intravenous/ intrafemoral or intraperitoneal injection of human CD34⁺ HSCs.	Multi-lineage reconstitution: T-cells, B-cells, NK cells, monocytes, DCs and Neutrophils. (Better reconstitution with knock-in strains).	• Stable and long-term multi-lineage engraftment including the myeloid compartment. • GVHD is less common, permitting long-term ( > 2 months) studies compatible with GBM. • Mucosal engraftment.	• Not all subsets are functionally developed. • Long time needed for differentiation ( > 10 weeks). • High cost. • HLA-restricted responses are impaired. • B-cells remain largely immature CD5⁺. • NK-cell and T-cell function is partially defective.	• Long-term efficacy studies. • Myeloid-focused studies.
Hu-BLT	Sublethal irradiation, renal subcapsular implantation of autologous human fetal thymus and liver, plus intravenous/ intraperitoneal autologous fetal liver-derived CD34⁺ HSCs.	• Multi-lineage reconstitution with HLA-restricted, thymically educated T-cells. • Stable Engraftment of multi-lineage T-cells, B-cells, DCs, macrophages and myeloid cells in addition to diverse T-cell subsets such as CD4^+^, CD8^+^ and Treg cells.	• The most complete reconstitution currently available • Authentic human thymic education enables genuine HLA-restricted antigen-specific responses. • The best available system for antigen-specific and vaccine questions. • Mucosal engraftment.	• Requires survival surgery and specialist expertise. • Ethically and legally restricted availability of human fetal tissue. • Higher GVHD incidence and shortened lifespan relative to Hu-CD34⁺. • High cost. • Long time needed for differentiation ( > 10 weeks).	• Antigen-specific and vaccine studies. • Mechanistic studies in which HLA restriction is essential.

^*^:(unpublished Preprint), **:(conference abstract).

*DC* Dendritic cells, *NK cells* Natural killer cells, *PBMC* Peripheral blood mononuclear cell, *HSC* hematopoietic stem cell, *Treg* Regulatory T cell, *PDX* Patient-derived xenograft.

Compiled from references [2, 21, 23, 41–43].

*DC* Dendritic cells, *NK cells* Natural killer cells, *PBMCs* Peripheral blood mononuclear cells, *HLA* human leukocyte antigen, *GVHD* Graft-versus-host disease, *GBM* Glioblastoma, *TME* Tumor microenvironment, *HSC* hematopoietic stem cell, *Treg* Regulatory T cell.

## Gut microbiome as an emerging modifier of humanised GBM models

Accumulating evidence has revealed a significant role for the gut microbiome in the initiation and progression of various diseases, such as metabolic diseases, liver diseases and cancer [51]. In the context of GBM, researchers have sought to evaluate the impact of the gut microbiome using HUM mice. HUM mice are typically generated by transplanting faecal material from healthy human donors into germ-free or gnotobiotic mice. In one such study, HUM mice were intracranially implanted with GL261 cells and subsequently treated with either an anti-PD-1 antibody or an isotype control. Sequencing of mouse faecal samples revealed differences in microbial diversity and composition among individual HUM mice. Notably, each mouse harbouring a distinct microbiome exhibited a different response to anti-PD-1 therapy [19]. In these methods, the only variable between animals is the composition of the gut microbiome.

Preliminary data presented in conference abstract form from the same programme additionally suggested that two distinct HUM mouse models established via faecal transplantation similarly showed divergent responses to both temozolomide and anti-PD-1 treatment. Furthermore, these models differed in the CD8^+^/Treg ratio in the spleen and in the frequency of cytotoxic CD8^+^ T-cells producing interferon-γ (IFN-γ) following PD-1 therapy, underscoring the critical role of gut microbiome humanisation in GBM research [52]. In another investigation, faecal samples from healthy controls and GBM patients were collected to compare the effect of the human gut microbiome on glioma development in mice. The results showed that the gut microbiome derived from high-grade glioma patients promotes glioma progression in mice, potentially via sphingosine 1-phosphate mechanisms [53].

The influence of the gut microbiome extends beyond tumour progression. A study using HUM mice showed that the gut microbiome modulates both the presence and gene activation profiles of immune cells in the GBM mouse models, under both control conditions and anti-PD-1 treatment [54]. Moreover, emerging evidence indicates that the gut microbiome undergoes changes during different stages of GBM growth and throughout temozolomide therapy, highlighting the importance of considering gastrointestinal microbiota in immune modulation, GBM promotion and treatment response [55, 56]. Collectively, these findings support the proposal that accurate recapitulation of the human gut microbiome should be integrated into current humanisation protocols for preclinical GBM models.

### Dual humanised models: integrating human immunity and the microbiome

Double humanised mouse models represent an advanced preclinical platform that simultaneously incorporates both human immune system components and the human microbiome into murine hosts [57]. The establishment of these dual humanised models typically follows a sequential approach, beginning with the development of a humanised immune system, followed by antibiotic-mediated depletion of the native murine microbiota (in non-germ-free settings) and subsequent transplantation of human faecal microbiota [58]. As discussed before, the human gut microbiome plays important roles in different physiological and pathological processes, particularly in the context of GBM. Here, we will discuss the double humanised mouse models, which offer considerable promise for in vivo investigations in neuro-oncology research.

### Germ removal using antibiotic treatment

In order to establish the double humanised mouse models, Lance Daharsh et al. employed female NSG mice (NOD.Cg-Prkdc^scid^Il2rg^tm1Wjl^/SzJ) and irradiated them with a dose of 12 cGy per gram of body weight. Subsequently, mice were implanted with a single human fetal thymic tissue and two human fetal liver fragments under the renal capsule, followed within 6 hours after surgery by intravenous administration of 1.5–5 × 10^5^ CD34^+^ HSCs. Following confirmation of successful humanisation by flow cytometry analysis, the mice underwent antibiotic-induced microbiota depletion with a cocktail of broad-spectrum antibiotics, including Ampicillin(1 g/L), Metronidazole(1 g/L), Neomycin(1 g/L) and Vancomycin(0.5 g/L), administered through the drinking water. Human faecal microbiota transplantation was then performed via oral gavage at 24 and 48 h following antibiotic treatment. The microbiome diversity of faecal samples was then characterised using 16S rRNA gene sequencing in the double humanised mouse, demonstrating a distinct human-like microbiome characteristic compared to the conventional humanised model lacking a human microbiome. Importantly, this study also established that the human-like microbiome remained stable for a duration of 14.5 weeks post-transplantation [59], supporting the utility of this approach for longitudinal investigations.

### Germ-free models

In another study, the gnotobiotic and germ-free NOG mice were used to establish the double humanised model. Following irradiation, the mice received the HSCs through intravenous injection. Subsequently, human faecal microbiota transplantation was performed to establish the human microbiome. This study suggested that a humanised microbiome promoted the maintenance and proliferation of the human CD3^+^ T cells in the humanised immune system mouse models [60].

Several important considerations must be addressed when employing double humanised models for biomedical research. Theoretically, these models represent among the most physiologically relevant platforms for simulating the gut microbiome and immune system interactions. However, it should be considered that the host immune system may influence the faecal microbiota transplantation (FMT) outcomes through species-specific immune selection mechanisms. Conversely, the transplanted human microbiome exerts immunomodulatory effects on the engrafted human immune system. Moreover, the timing of transplantation and antibiotic treatment in non-germ-free models can significantly affect engraftment efficiency and stability [61]. Nevertheless, given the executive roles of the microbiome in various pathological conditions, especially GBM [19, 54, 56, 62], current evidence suggests that double humanised models can recapitulate human immune system-gut microbiome interactions at an acceptable rate [57].

## Genetically humanised strains

In this section, we briefly discuss genetically humanised mouse models and their applications in GBM research. In most cases, genetically engineered models of GBM are predominantly employed to evaluate the roles of specific genes in tumour initiation and progression [63]. Genetic humanisation is an evolving field that integrates human DNA sequences into the mouse genome to more accurately model human diseases. Genetic humanisation is accomplished through the following main approaches: (1) homologous recombination and recombinase-mediated cassette exchange in embryonic stem cells, which have been applied to loci up to 200 kb; (2) CRISPR/Cas9-associated homologous recombination using short or long ssODN donors; and (3) ssODN-mediated end joining in zygotes [64]. These strategies have expanded the potential for humanisation, improved immune cell engraftment and established more efficient and stable human immune system reconstitution.

For example, Billerbeck et al. developed a genetically engineered mouse model expressing the stem cell factor (SCF), granulocyte–macrophage colony-stimulating factor (GM-CSF) and interleukin (IL)-3, resulting in superior engraftment and differentiation of human immune cells following immune system humanisation [65]. Another study used Velocigene technology to establish a human Thrombopoietin (TPO) knock-in in the RAG2^−/−^γc^−/−^ mouse background, demonstrating improved engraftment and multi-lineage differentiation [66].

Genetic modification can also facilitate serial engraftment across different mouse strains and support long-term haematopoietic stem cell transplantation. For instance, knock-in of four human cytokines (M-CSF, IL-3, GM-CSF and thrombopoietin) into the MISTRG strain using Velocigene technology enables serial engraftment for up to four generations. This genetic configuration supports the development of most human haematopoietic cells in mice, as well as tissue macrophages, NK cells, dendritic cells and lymphocytes [67, 68].

CRISPR-Cas9 has emerged as a powerful genome-editing technology, increasing the flexibility and throughput of genetically humanised mouse models. CRISPR technology enables precise single-locus and multiplex targeted genome editing, facilitating the expansion of genomic humanisation strategies in mice. Recent studies demonstrate that CRISPR-Cas9 can be integrated with large-fragment humanisation methodologies (e.g. bacterial artificial chromosome (BAC)-based targeting) to facilitate replacement of extended mouse loci with corresponding human orthologous genomic fragments while preserving important regulatory regions. Importantly, humanised alleles generated by CRISPR-Cas9 editing retain their human-like splicing isoforms and organ-specific expression patterns, providing a versatile, accurate and disease-oriented platform for modelling human gene function [64, 69]. In this context, CRISPR has become an indispensable tool for somatic GBM modelling and in vivo screening. Unlike traditional, time-consuming germ-line breeding strategies, CRISPR-Cas9 genomic engineering enables rapid, precise deletion of multiple tumour suppressor genes (*Trp53, Pten* and *Nf1*), generating GBM in the mouse brain. When combined with the replication-competent avian leukosis virus splice-acceptor (RCAS-TVA) system, this approach permits cell-type-directed brain tumour modelling through somatic deletion of specific genes including *Trp53*, *Cdkn2a* and *Pten*, as well as the *Bcan-Ntrk1* fusion in neural stem cells [70, 71].

Furthermore, the versatility of CRISPR technology enables direct in vivo stereotactic intracranial injection of an adeno-associated virus (AAV) CRISPR library into conditional-Cas9 mice, thereby generating tumours that mirror key genetic features of human GBM [72]. By combining in utero electroporation with CRISPR-Cas9 genomic editing, an immunocompetent mouse glioma model has been established. Based on transcriptomic analyses, the model has been instrumental in characterising vascular and endothelial heterogeneity during glioma progression, with the distinct molecular signatures identified in these endothelial populations also detected in human glioma specimens [73].

Importantly, it is crucial to distinguish between traditional humanised mouse models, generated through engraftment of human immune cells or tissues and CRISPR GBM models, which are primarily designed for genome editing and mechanistic tumour studies. At present, most CRISPR-based GBM functional studies have not been performed within the standard immune-humanised platforms discussed in this review. Instead, they have mainly relied on mouse-native glioma models or genetically engineered tumour models rather than Hu-PBL, Hu-CD34^+^, or Hu-BLT platforms. A promising future direction would be to combine CRISPR-based genomic editing with human immune cell engraftment within the same model, potentially enabling more comprehensive investigations of tumour-immune interactions in a humanised immunological context. It is noteworthy that Genetic humanisation raises ethical and regulatory considerations because human sequences are integrated into the germline, therefore modifications and any associated welfare burden will be transmitted to the subsequent generation which requires prospective severity classification, harm benefit assessment, continuous welfare monitoring and clear humane endpoints under regulations such as EU Directive 2010/63/EU and equivalent national legislation, alongside strict application of the 3Rs and transparent reporting in accordance with ARRIVE 2.0 guideline [74, 75].

## Host strain selection for humanised GBM modelling

The choice of host strain is a critical determinant of successful human immune system engraftment and subsequent GBM modelling. A comparative study evaluated the occurrence of post-engraftment symptoms following transplantation of human CD34^+^ HSCs in two commonly used humanisation strains (NSG-SGM3 and NOG-EXL) for the establishment of orthotopic PDX models of intracranial GBM. The results indicated that MHC syndrome (elevated leukocytes, widespread tissue lesions) was more frequent in NSG-SGM3 mice, limiting long-term GBM studies, with extensive mast-cell infiltration of the pancreas and hepatocellular necrosis [76]. In a separate study, humanised NSG-SGM3 mice consistently developed lethal MHC syndrome prior to tumour transplantation, regardless of whether the HSCs were of autologous or allogeneic origin [77].

**Fig. 3:**
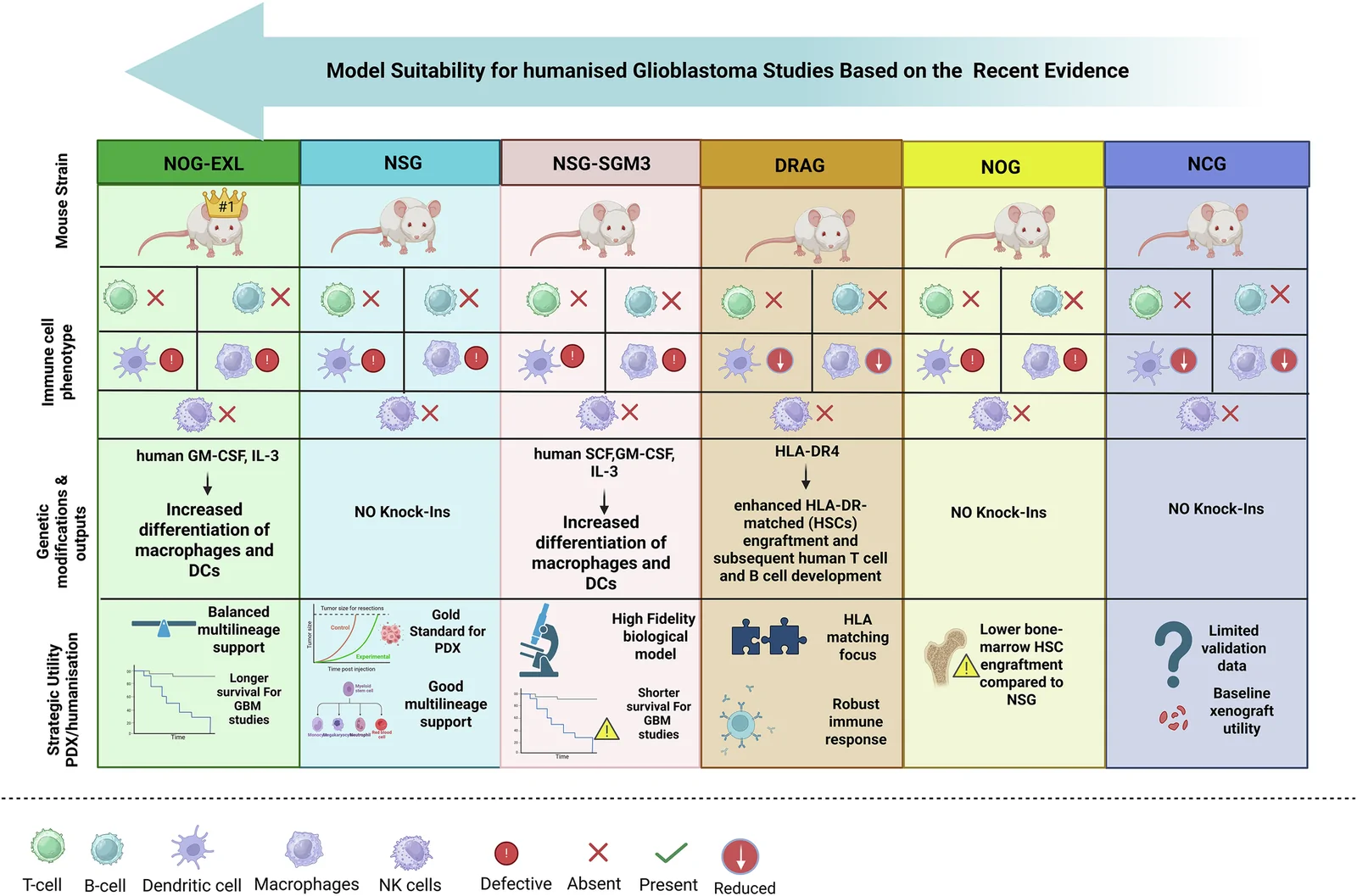
Different strains used for the humanised PDOXs in GBM research. Considering the survival time as an important factor in GBM research and also the ability for multi-lineage immune system engraftment and patient tissue engraftment, the different mouse strains are set in an order based on the available published data. NOG-EXL offers better characteristics compared to others. It has the human GM-CSF and IL-3 knock-ins, which help to increase and improve differentiation of macrophages and DCs compared to other strains; abbreviations: GM-CSF, granulocyte-macrophage colony-stimulating factor; SCF, Stem Cell Factor; HSCs, hematopoietic stem cells; HLA, human leukocyte antigen. Created in BioRender.com [21, 23, 78, 79, 107, 108].

Another comparative analysis examined NSG-SGM3, NOG-EXL, NCG and NSG strains. NSG-SGM3 mice showed higher levels of human monocytes, mast cells, T-cells, DCs and megakaryocytes, along with low circulating platelet counts. NOG-EXL mice exhibited a similar cellular profile but with more inactive circulating platelets. In contrast, NSG and NCG mice developed lower overall numbers of human immune cells [78]. Furthermore, direct comparison between NSG and NOG strains revealed that NSG supports higher levels of humanisation [79]. However, another study reported that NOG-EXL mice achieve higher humanisation levels than NSG-SGM3 [42]. Based on the current available data, the NOG-EXL strain appears particularly well-suited for studies requiring strong myeloid lineage engraftment and long-term experimental stability. Nevertheless, optimal strain selection remains highly dependent on the specific endpoints of each study (Fig. 3).

## Practical design considerations for humanised GBM models

### Preconditioning and irradiation strategy

In order to establish the humanised mouse models (Hu-CD34^+^, Hu-BLT), sublethal irradiation is essential to ablate the murine immune system cells and create a receptive niche for human HSC engraftment [2]. For the Hu-PBL model, irradiation is not recommended, especially if a long-term study like that for GBM research is needed, as irradiation promotes more CD4^+^T cell engraftment and subsequently a higher rate of GVHD occurrence [27]. The dose of sublethal irradiation is an important consideration, as some mouse strains can tolerate the irradiation at a moderate level, but some are sensitive. For example, mouse strains with Prkdc mutations are sensitive to irradiation due to defective DNA double-strand break repair [80]. Conversely, strains with Rag1/Rag2 mutations demonstrate greater resistance to irradiation and can tolerate higher doses [81]. As mentioned before, the inoculation of pups with immune cells will lead to higher levels of T cell engraftment. Studies in mice possessing the Prkdc mutation, such as the NSG, NOG, NCG and MITRG /MISTRG, have reported tolerance dosages of 100 cGy,75 cGy, 70 cGy and 150 cGy, respectively. Also, mice with Rag mutations, such as the BRG and BRGS/BRGST, can tolerate doses of 3.3 Gy and 3 Gy, respectively [81]. For older (5–12 weeks old) NSG mice, doses of 240 cGy are commonly employed, with reported ranges of 50–250 cGy depending on age and ^137^Cs source, although some studies indicate tolerance up to 400 cGy [24, 82]. Other studies have used varying irradiation doses in different strains. For example, in order to establish the Hu-CD34^+^ model, researchers used 1.4 Gy for 3-week-old NSG, 1.1 Gy for 3-4-week-old NOG and 1 Gy for 5-week-old NSG-SGM3 [83]. Moreover, the NOG-EXL strain has been successfully irradiated at 1.5 Gy [84]. For GBM research specifically, irradiation doses in humanised mouse models range from 90 cGy to 2.5 Gy (Table 1A), with the precise dose determined by strain sensitivity and animal age. It is recommended that initial testing for Prkdc-mutant strains commence within the 60-110 cGy range, while Rag-mutant strains can be tested starting from 2 Gy to 4 Gy [81].

The C57BL/6 strain, commonly utilised in syngeneic GBM studies, exhibits significantly greater radiation resistance compared to immunodeficient strains with Prkdc mutations [85]. Interestingly, strains harbouring Kit mutations eliminate the requirement for irradiation. More specifically, a study conducted on the NSG strain with the Kit^W41J^ mutation (NSG-W41) demonstrated successful engraftment of CD34^+^HSCs without preconditioning irradiation, while additionally supporting enhanced human myeloid differentiation, erythropoiesis and thrombopoiesis [86, 87]. Furthermore, crossing C57BL/6J-Kit^W41J^ mice with the NSG strain generated the non-albino NBSGW mice, which achieve successful engraftment without radiation and exhibit superior humanisation compared to conventional NSG mice [88]. Ultimately, these models offer significant advantages by eliminating irradiation-associated toxicity and simplifying experimental protocols.

### Cell source, dose and engraftment logistics

In order to have good engraftment, the HSCs and the PBMCs should be inoculated at a suitable number of cells. For GBM research applications, studies have employed HSC doses ranging from 10^4^ to 2 × 10^5^ cells, while PBMC doses are consistently reported at 10⁷ cells (Table 1A). Based on comprehensive analyses, the optimal CD34⁺ HSC dose is recommended at 3 × 10⁴ to 10⁵ cells [24, 89]. For PBMCs, a minimum of 5 × 10⁶ cells and an optimal range of 5–10 × 10⁶ T cell-containing PBMCs are required for stable engraftment [83]. Some studies have indicated that administration of up to 3 × 10^7^ PBMCs correlates with increased humanisation levels, although 2 × 10^7^ is recommended to mitigate GVHD risk [90]. Moreover, a positive correlation has been reported between humanisation (hCD45^+^ levels in peripheral blood) and PBMC dose, with GVHD risk similarly correlated with PBMC dose [83]. HSC and PBMC reconstitution show donor-dependent variability; additionally, humanised mice from different donors respond differently to immune checkpoint inhibitors [91, 92]. To minimise this, mice from at least two donors should be randomly assigned per group, enabling robust comparisons and reducing donor-specific effects. In order to increase and yield higher numbers of HSCs, in vitro expansion represents a potential approach; however, the expansion capacity is limited to a 10-20 fold increase and in addition, the expansion protocols may result in the loss of characteristic stemness properties [93].

### Cryopreserved versus fresh human cells

The source of human cells—whether fresh or cryopreserved—constitutes an important logistical consideration with potential implications for engraftment efficiency. A study demonstrated that HSCs from umbilical cord blood cryopreserved for 27 years maintained robust engraftment capacity in NSG mice, although a low engraftment rate (33%) was observed in the oldest unit, consistent with the 10–20% failure rate encountered in clinical cord blood transplantation. Additionally, RNA-seq analysis revealed transcriptional distinctions between fresh and cryopreserved samples, but cryopreservation did not alter pathways associated with HSC function [94]. Also, another study showed that engraftment success rates are not correlated with cryopreservation status [95].

However, comparative studies have reported modestly higher humanisation levels with fresh CD34^+^ HSCs, with fresh cells yielding hCD45^+^ levels of 20.8% compared to11% for cryopreserved samples [96]. Moreover, cryopreservation has been shown to have significant effects on the number and viability of PBMCs, with T lymphocytes, particularly CD4^+^ T cells, being most affected. Notably, different lymphocyte subtypes exhibit varying tolerance to cryopreservation [97]. Despite these effects, reconstitution efficacy using cryopreserved versus fresh PBMCs from identical donors showed no obvious differences, with humanisation requirements met in 6/6 fresh and 5/6 cryopreserved samples [90].

### HLA matching and autologous versus allogeneic design

The HLA are proteins essential for antigen and neoantigen presentation to downstream immune effector cells. There are three different classes of HLA: (i) class I, which encompasses the A, B and C alleles, (ii) class II, which includes the DP, DQ and DR alleles and (iii) class III, which includes the genes implicated in inflammatory responses, leukocyte maturation and the complement cascade [98].

HLA Matching between tumours and implanted immune cells is particularly important for inducing tumour antigen-specific T cell responses. Although a study investigating HLA mismatch between PDX samples and CD34^+^ HSCs found no effect on tumour growth kinetics, it was observed that similarity in multiple HLA class I and II loci in tumour samples and immune cells correlates with reduced tumour growth due to stronger autologous reactions [91]. Another study reported that matching of HLA class I and II serotypes between PBMCs and tumour samples may impede tumour growth through autologous reactions, suggesting T cell recognition of HLA proteins even with low expression levels. This phenomenon highlights the importance of using autologous PBMCs from the same tumour tissues to achieve high-precision research and advance towards personalised medicine [90].

Another study showed that PDX tumour growth is slower in the humanised mouse compared to the non-humanised model, although the differences were not significant, underscoring that HLA matching may not have a significant effect on the tumour growth rate [99]. Interestingly, a study using hepatocellular carcinoma PDX models demonstrated significantly increased tumour growth in the presence of human immune cells compared to non-humanised controls, with HLA typing revealing that a minimum of two out of four alleles matched on HLA-A and HLA-B between PDX and HSCs [100]. Other investigations found no correlation between HLA matching and humanisation extent (assessed by hCD45^+^ tumour infiltration) or between HLA matching and anti-tumour responses, suggesting that HLA matching may not be universally required for effective anti-tumour immunity [101]. However, data reported in conference abstract form indicated that when HLA matching was considered at the PDX-PBMC level with 33–100% compatibility across class I and II HLA profiles, tumour growth was more effectively inhibited in HLA-matched profile models following treatment with immune checkpoint inhibitors [102].

A fundamental distinction in the design of humanised mouse models for GBM research lies in the relationship between the source of human immune cells and the tumour tissue implanted. In autologous models, both immune cells and PDX samples are from one patient, whereas in allogeneic models, we use the immune cells from different donors [2].

Autologous models reliably achieve tumour engraftment while preserving HLA-restricted, antigen-specific T-cell responses; also, these models more closely mirror the parental tumour and treatment responses [103, 104].

Within this context the studies reviewed here employed a wide spectrum of matching between the tumor samples and immune cells, for instance: HLA class I loci matched (HLA-A, HLA-B) and variable matching across HLA class II loci(HLA - DP,-DQ and -DR [41] mismatched samples [42], HLA class I matched (HLA-A2)[45] autologous tumour immune cells pairs [46] multiple matched HLA class I loci (HLA-A, -B and -C) and class II loci (HLA-DRB1 and HLA-DQB1) [47, 48] and finally matched only on HLA class I loci (HLA-A) [50].

In summary, published HLA-matching strategies are heterogeneous, from single-locus class I to multi-locus class I/II. This variability influences tumour growth and engraftment via allogeneic/autologous reactions and should be considered when selecting or interpreting these models, especially where HLA-restricted antigen presentation is functionally relevant.

## Limitations of humanised models and strategies to overcome them

Despite their numerous advantages, humanised mouse models for GBM research are associated with several inherent limitations. These include: the requirement for a high number of cells to achieve robust engraftment in the Hu-CD34^+^ model; limited accessibility of human fetal tissues for the Hu-BLT model; prolonged differentiation time required for both the Hu-CD34^+^ and Hu-BLT models; restricted development of certain human immune cell lineages; occurrence of GVHD in the Hu-PBL model; lack of species-specific growth factors, cytokines and chemokines; and MHC mismatch between human immune cells and the murine host [2, 21, 23]. To address these challenges, several strategies have been developed. To reduce the risk of GVHD, particularly in the Hu-PBL model, approaches include injecting a lower number of PBMCs (1-5 ×10^6) and utilising MHC KO strains such as HUMAMICE, NOG-dKO(NOG-B2M^null^IA^null^), NSG-B2M^null^, NSG-B2M^null^(IA IE)^null^ and NSG-MHC I/II DKO, NSG-(K^b^D^b^)^null^ IA^null^ [2, 23].

To overcome the limited development of innate immune cell populations in both Hu-PBL and Hu-CD34^+^ models, knock-in mouse strains have been developed to enhance specific lineages: NOG-EXL, NSG-SGM3, MITRG, MISTRG and MISTRG6 promote improved myeloid lineage development; NSG-Tg [Hu-IL15], hIL2-NOG and MISTRG support better NK development; and the BRGST strain facilitates enhanced B-cell development. Also in the Hu-CD34^+^ model, murine thymic education limits the development of HLA-restricted T cells, a limitation now addressed by the HLA transgenic strains. These strains may express either the Class Ι or II HLA or both of them, including different alleles like: DRAG (NRG-DR4, NRG/HLA-DR4Tg), DRAGA (NRG-A2/DR4, NRG/HLA-A2Tg, HLA-DR4Tg), HUMAMICE(B6RG/B2M–/–, IAβ–/–, Perf–/–, HLA-A2Tg, HLA-DR1Tg) [2, 23, 105, 106].

## Conclusions and future directions

Mouse models remain an indispensable cornerstone of preclinical research, especially in the field of GBM. They are essential for recapitulating the complex architecture of the blood-brain barrier and the multifaceted interactions between the TME and various organ systems. Although conventional mouse models continue to offer significant utility, their inherent limitations have driven the development of more specialised and advanced platforms, including humanised mouse models.

Humanised PDX models faithfully recapitulate the core immunological features of the human GBM and its TME, as well as the histopathological and molecular characteristics observed in GBM patients. By integrating tumour molecular profiles, human immune components and physiological properties of the human organs, humanised PDX mouse models provide an invaluable platform for the rational design and preclinical evaluation of immunotherapies and personalised treatment strategies for GBM. As discussed before, in humanised mouse models the human immune system compartments are reconstituted and the brain-specific barriers such as BBB, BCSFB and BMB remain murine; however, these barriers can be simulated at an acceptable rate for the advancement of preclinical drug development studies.

In the present study, we also discussed an often overlooked factor in humanised modelling: the gut microbiota and its role in immune modulation, particularly in the context of GBM immuno-oncology. Accumulating evidence suggests that incorporating the human microbiome into humanised mouse models may represent a critical step toward improving the translational relevance of GBM research. A humanised microbiome contributes to the GBM TME in three different ways: first, microbial metabolites can directly modulate the immune compartments of the TME, such as microglial cells, which play an important role in shaping the tissue-associated macrophage population as the dominant resident cells in the TME. Second, the composition of the microbiota determines the response to immunotherapies. HUM mice carrying different microbiota showed divergent benefits from anti-PD-1 as well as distinct intratumoral immune cell activation states. Third, the microbiota also influences cytotoxic chemotherapy, since gut microbial composition changes during temozolomide treatment and the microbiota modulates temozolomide efficacy through immunomodulation. Additionally, microbiota from patients with high-grade glioma accelerate glioma progression in recipient mice.

Incorporating a human microbiome, therefore, will allow the human immune compartment, the human tumor and the microbial metabolite milieu that influences both to be studied in a single animal and would open the way to testing dietary, probiotic, or FMT-based interventions as adjuvants to the immunotherapies.

Future efforts should therefore focus on the development of next‑generation humanised models that integrate not only human immune system components but also a humanised gut microbiome, thereby enabling more accurate prediction of therapeutic responses and resistance mechanisms in GBM.

## Data Availability

Data sharing is not applicable to this article as no new datasets were generated or analysed. All data discussed are available in the cited published literature.
